# Mediating role of accelerated aging in the association between depression and mortality risk: findings from NHANES

**DOI:** 10.1007/s40520-024-02854-z

**Published:** 2024-10-05

**Authors:** Cheng Xu, Jia-ni Wang, Zhen Song, Han-yu Deng, Chong-chao Li

**Affiliations:** 1 Nanjing University of Chinese Medicine, 138 Xianlin Avenue, Nanjing, Jiangsu 210023 China; 2 Yancheng Binhai Hospital of Traditional Chinese Medicine, Yancheng, China; 3 Institute of Acupuncture and Moxibustion, China Academy of Chinese Medical Sciences, Beijing, China

**Keywords:** Depression, Accelerated aging, Mortality, National health and nutrition examination

## Abstract

**Objective:**

To investigate the association between depression, accelerated biological aging, and mortality risk, and to assess whether accelerated aging mediates the relationship between major depression and mortality risk.

**Methods:**

A prospective cohort of 12,761 participants aged 20 years or older from the 2005–2010 cycle of the National Health and Nutrition Examination Survey (NHANES) was analyzed. Depression was assessed using the Patient Health Questionnaire-9 (PHQ-9), with scores of ≥ 10 indicating major depression. Accelerated biological aging was measured using phenotypic age acceleration (PhenoAgeAccel). Multivariable linear regression models and subgroup analyses were used to examine the association between depression and accelerated aging, while weighted multivariable Cox proportional hazards regression models and subgroup analyses assessed the impact of major depression on mortality risk. Mediation analysis was performed to assess whether PhenoAgeAccel mediated the relationship between major depression and mortality outcomes.

**Results:**

Among the 12,761 adults, the weighted mean age was 46.6 years, with 48.8% being male, and 6.9% experiencing major depression. The results showed a positive association between major depression and PhenoAgeAccel (β: 0.61, 95% CI: 0.06–1.16). Over a median follow-up duration of 11.3 years (interquartile range: 9.9–13.1), major depression was associated with increased all-cause mortality (HR: 1.35, 95% CI: 1.13–1.62) and cardiovascular mortality (HR: 1.73, 95% CI: 1.18–2.54). However, the relationship with cancer mortality was not statistically significant after full adjustment for confounding factors. The mediation analysis further revealed that PhenoAgeAccel accounted for 10.32% and 5.12% of the associations between major depression and all-cause mortality, and cardiovascular mortality, respectively.

**Conclusion:**

Depression is associated with accelerated aging and contributes to increased all-cause and cardiovascular mortality. Accelerated aging partially mediates the association between major depression and mortality risk. Our findings highlight the urgent need to incorporate mental health care into public health strategies to delay population aging and reduce mortality risk.

**Supplementary Information:**

The online version contains supplementary material available at 10.1007/s40520-024-02854-z.

## Introduction

Depression is a prevalent mental disorder characterized by a diminished interest in daily activities, insomnia, anhedonia, and suicidal ideation, significantly impairing psychosocial functioning and quality of life [1–3]. It poses a significant global mental health challenge, with the number of incident cases of depression worldwide rising from 172 million in 1990 to 258 million in 2017, representing a 49.86% increase [4, 5]. Depression is particularly common in older people due to factors such as chronic diseases, physical disability, and social isolation [6, 7]. The global prevalence of major depression in older adults was 13.3% [8]. However, due to the frequent coexistence with physical comorbidities and cognitive impairment, depression in older people is often underdiagnosed, which may lead to an underestimation of its true prevalence [9]. Depression can elevate the risk for aging-related diseases, and exacerbate the severity of various medical conditions and even increase mortality rates [10–14]. A large cohort study using UK Biobank data has also demonstrated a significant association between accelerated biological aging and depression [15]. Given the substantial burden of depression and the challenges of an aging population, preventive strategies focusing on modifiable risk factors are urgently needed.

Biological aging refers to the progressive decline in system integrity that accompanies advancing age, ultimately resulting in disease and death [16, 17]. Unlike chronological age, which merely indicates the time elapsed, biological age provides a more precise measure of aging status by considering health condition [18]. Biomarkers of aging, essential for assessing biological age, are generally categorized into omics data, such as DNA methylation [19], metabolomics [20], and proteomics [21], and clinical biomarkers obtained from blood chemistry, hematology, anthropometry, and organ function tests [22–24]. Among these biomarkers, algorithms that integrate standard clinical parameters have demonstrated high accuracy in predicting morbidity and mortality [25], in addition to being more economically feasible. A novel measure of biological aging, phenotypic age acceleration (PhenoAgeAccel), has been developed based on multisystem clinical biomarkers [26, 27]. This measure captures whole-body aging and assesses morbidity and mortality risk using data from the National Health and Nutrition Examination Surveys (NHANES), making it suitable for evaluating aging interventions and identifying high-risk groups for death and disease in the United States (U.S.) [26, 27].

Existing research on the impact of depression on biological aging and mortality risk remains inconclusive. This study uses data from the NHANES, a large and representative dataset of the U.S. population, to evaluate the association of depression with accelerated aging and mortality risk among U.S. adults, and further explore the potential mediating role of accelerated aging in the relationship between depression and mortality outcomes. The findings are expected to inform the optimization of public health strategies, thereby alleviating the societal burden caused by depression.

## Methods

The NHANES is a national population-based study in the U.S. that has collected data on the health and nutritional status of non-institutionalized U.S. civilians every two years since 1999. Participants were selected through a complex multistage probability sampling process, ensuring that the results are generalizable to the entire U.S. population. All NHANES protocols were approved by the National Center for Health Statistics ethics review board, and written informed consent was obtained from all participants.

### Study population

Each participant was invited to complete an in-person interview and undergo a series of physical examinations and laboratory tests at a mobile examination center. This study combined the NHANES continuous datasets from 2005-2006 to 2009-2010. Data from participants in these NHANES cycles were linked to their mortality outcomes. Among the 31,034 participants, individuals without depression questionnaire data (*n* = 15,274), those missing blood biomarker data (*n* = 895), those aged below 20 years (*n* = 956), or lacking relevant covariate information (*n* = 1,148) were excluded from the analysis. Consequently, 12,761 participants were included in the primary analysis to investigate the relationship between depression and accelerated aging. However, due to insufficient identifying data, eight participants were not eligible for mortality linkage, resulting in 12,753 participants being analyzed for the association between depression and mortality outcomes (Supplementary Fig. 1).

### Assessment of depression

The Patient Health Questionnaire-9 (PHQ-9) was used to diagnose and assess the severity of depressive symptoms. The PHQ-9 consists of nine questions, each scored from 0 to 3, resulting in a total score ranging from 0 to 27. Higher scores indicate more severe depressive symptoms. Each question is scored from “0” (not at all) to “3” (nearly every day). A total score of ≥ 10 was classified as major depression [28]. To explore different levels of depressive symptoms, scores were categorized as follows: 0 to 4 indicated no depressive symptoms, 5 to 9 mild depressive symptoms, 10 to 14 moderate depressive symptoms, and ≥ 15 severe depressive symptoms [29].

### Assessment of phenoAgeAccel

A new phenotypic age (PhenoAge) metric, rather than chronological age alone, could more accurately predict health-related outcomes. This measure was previously developed and validated in the U.S. population [26, 27]. Following the PhenoAge definition, we calculated PhenoAge using ten aging-related variables: chronological age, albumin, creatinine, glucose, C-reactive protein, lymphocyte percent, mean cell volume, red blood cell distribution width, alkaline phosphatase, and white blood cell count. To account for the effect of chronological age, we defined PhenoAgeAccel by extracting residuals from regressing PhenoAge on chronological age (Supplementary Methods 1). Positive PhenoAgeAccel values indicate an older physiological age, while negative values indicate a younger physiological age.

### Assessment of mortality

To determine mortality status during follow-up, we used the NHANES Public-Use Linked Mortality Files, which were matched to National Death Index records through December 31, 2019 (https://www.cdc.gov/nchs/data-linkage/mortality-public.htm). All-cause mortality was recorded as death from any cause. Specific causes of death were identified using the International Statistical Classification of Diseases, 10th Revision (ICD-10). Cardiovascular death included rheumatic heart diseases, hypertensive heart and renal disease, ischemic heart disease, and heart failure (ICD-10 codes I00–I09, I11, I13, and I20–I51). Cancer deaths were attributed to malignant neoplasms (ICD-10 codes C00–C97). The follow-up duration, measured in months, was calculated from the interview date to the date of death or December 31, 2019 for participants who remained alive at the end of the study period.

### Assessment of covariates

Sociodemographic and health-related behaviors, such as age, gender, race, education level, marital status, poverty income ratio, drinking status, smoking status, and physical activity were obtained through interviews. Body mass index (BMI), measured during physical exams, was calculated as weight in kilograms divided by height in meters squared. History of hypertension was identified by self-reported history, use of antihypertensive medication, or systolic blood pressure ≥ 130 mmHg or diastolic blood pressure ≥ 80 mmHg [30]. History of diabetes was identified by a self-reported doctor diagnosis, use of insulin or oral glucose-lowering drugs, plasma fasting glucose levels ≥ 126 mg/dL, random blood glucose or 2-hour oral glucose tolerance test blood glucose ≥ 200 mg/dL, or glycated hemoglobin A1c ≥ 6.5% [31]. History of cardiovascular disease (including congestive heart failure, coronary heart disease, angina, heart attack, and stroke) and cancer was self-reported by participants who had received diagnoses from a health professional.

### Statistical analysis

Based on NHANES analytical guidelines, sampling weights were applied to all analyses to interpret the complex survey design. Data were presented as the weighted mean with 95% confidence interval (CI) or weighted percentage with 95% CI. Baseline characteristics were compared using weighted linear regression for continuous variables and the Chi-square test for categorical variables. Multivariable linear regression models were employed to investigate the association between depression and PhenoAgeAccel (Supplementary Methods 2). Subgroup analyses and interaction tests were performed to explore whether the difference of associations between the subgroups. The subgroup factors included age, gender, race, poverty income ratio, BMI, physical activity, and the history of hypertension, diabetes, cardiovascular disease, and cancer. Survival analysis, including all-cause and cause-specific mortality, was conducted using weighted multivariable Cox proportional hazards regression models (Supplementary Methods 2). Subgroup analyses and interaction tests were conducted based on the history of hypertension, diabetes, cardiovascular disease, and cancer to assess whether the associations with all-cause, cardiovascular, and cancer mortality were consistent across these subgroups. Finally, mediation analysis was performed to assess whether the effect of major depression on mortality outcomes was mediated by the PhenoAgeAccel.

All statistical analyses were performed using R (http://www.r-project.org; version 4.4.0) and EmpowerStats (http://www.empowerstats.com; version 4.2). Statistical significance was set as a two-sided *P* < 0.05.

## Results

### Baseline characteristics

A total of 12,761 U.S. adults were included in this analysis. The weighted mean age was 46.6 years (95% CI: 45.9, 47.3), with 48.8% being male, and 6.9% of the participants experiencing major depression. Table 1 shows certain distinguishing characteristics between participants with and without major depression. Those with major depression tended to be younger, female, Non-Hispanic Black, less educated, less likely to be married, and had a lower poverty income ratio. They were also more likely to consume alcohol, smoke, engage in less physical activity, have a higher BMI, and have a history of hypertension, diabetes, cardiovascular disease, and cancer. Notably, participants with major depression exhibited higher levels of PhenoAge and PhenoAgeAccel, along with increased mortality rates.

**Table 1:** Basic characteristics of the study participants

Characteristics	No major depression	With major depression	*P* value
Age (years)	46.7 (46.0, 47.4)	45.6 (44.6, 46.6)	0.030
Age category (%)			< 0.001
20–39	37.2 (35.5 ,38.9)	35.9 (32.2 ,39.8)	
40–59	39.2 (37.9 ,40.5)	47.7 (43.4 ,52.0)	
≥60	23.6 (22.0 ,25.3)	16.4 (14.2 ,19.0)	
Gender (%)			< 0.001
Male	49.8 (49.0, 50.6)	35.8 (32.9, 38.9)	
Female	50.2 (49.4, 51.0)	64.2 (61.1, 67.1)	
Race (%)			< 0.001
Mexican American	7.9 (6.3, 9.9)	9.1 (6.3, 12.9)	
Other Hispanic	4.0 (3.0, 5.3)	6.2 (4.2, 9.0)	
Non-Hispanic White	73.1 (69.4, 76.5)	64.9 (58.7, 70.5)	
Non-Hispanic Black	9.8 (8.1, 11.8)	15.1 (12.2, 18.5)	
Other Race	5.3 (4.5, 6.2)	4.8 (3.4, 6.9)	
Education (%)			< 0.001
Less than high school	16.9 (15.4, 18.6)	28.9 (24.6, 33.6)	
High school	23.8 (22.5, 25.1)	27.7 (23.9, 31.7)	
More than high school	59.3 (56.8, 61.7)	43.5 (38.6, 48.4)	
Marital status (%)			< 0.001
Never married	15.9 (14.6, 17.5)	17.6 (14.9, 20.7)	
Widowed/divorced/separated	17.3 (16.4, 18.2)	31.0 (27.9, 34.3)	
Married/living with partner	66.8 (64.9, 68.6)	51.4 (47.9, 54.9)	
Poverty income ratio (%)			< 0.001
<1.3	17.3 (16.0, 18.7)	40.5 (36.6, 44.5)	
1.3 to < 3.5	35.9 (34.1, 37.8)	34.8 (31.2, 38.7)	
≥3.5	46.8 (44.3, 49.2)	24.7 (21.0, 28.8)	
Drinking status (%)			0.042
≥12 alcohol drinks per year	23.7 (21.9, 25.5)	26.6 (23.7, 29.6)	
<12 alcohol drinks per year	76.3 (74.5, 78.1)	73.4 (70.4, 76.3)	
Smoking status (%)			< 0.001
Never smoker	53.6 (51.8, 55.4)	38.5 (33.9, 43.2)	
Former smoker	25.5 (24.1, 26.9)	20.2 (16.9, 24.0)	
Current smoker	20.9 (19.6, 22.2)	41.3 (37.0, 45.7)	
Physical activity (%)			< 0.001
Inactive	45.8 (43.8, 47.7)	56.0 (52.1, 59.8)	
Moderate	27.4 (26.1, 28.7)	24.5 (21.6, 27.7)	
Vigorous	26.8 (25.4, 28.3)	19.5 (16.4, 23.0)	
BMI (%)			< 0.001
<25	31.5 (29.9, 33.1)	27.4 (23.6, 31.5)	
25 to < 30	34.1 (33.0, 35.3)	28.2 (25.2, 31.3)	
≥30	34.4 (32.8, 35.9)	44.5 (40.1, 48.9)	
Hypertension (%)			< 0.001
No	52.6 (51.1, 54.1)	44.2 (40.5, 48.0)	
Yes	47.4 (45.9, 48.9)	55.8 (52.0, 59.5)	
Diabetes (%)			< 0.001
No	88.4 (87.5, 89.3)	81.1 (77.7, 84.0)	
Yes	11.6 (10.7, 12.5)	18.9 (16.0, 22.3)	
Cardiovascular disease (%)			< 0.001
No	92.6 (91.9, 93.3)	84.7 (81.9, 87.2)	
Yes	7.4 (6.7, 8.1)	15.3 (12.8, 18.1)	
Cancer (%)			0.001
No	91.3 (90.5, 92.1)	87.6 (84.7, 90.0)	
Yes	8.7 (7.9, 9.5)	12.4 (10.0, 15.3)	
PhenoAge (years)	39.1 (38.3, 40.0)	40.4 (39.1, 41.7)	0.048
PhenoAgeAccel (years)	-7.6 (-7.9, -7.2)	-5.2 (-6.1, -4.4)	< 0.001
Mortality outcomes ^a^ (%)			0.002
No	88.9 (87.7 ,89.9)	84.9 (82.1 ,87.3)	
Yes	11.1 (10.1 ,12.3)	15.1 (12.7 ,17.9)	

Data were presented as weighted means or percentages (95% confidence intervals)

Abbreviations: BMI, Body mass index, PhenoAge, phenotypic age; PhenoAgeAccel, phenotypic age acceleration

^a^ Eight participants were not linkage eligible due to having insufficient identifying data to mortality status

### Association between depression and PhenoAgeAccel

Weighted multivariable linear regression models were used to investigate the association between depression and PhenoAgeAccel. As shown in Table 2, the analyses revealed a positive association between major depression and PhenoAgeAccel in the unadjusted model (β: 2.04, 95% CI: 1.48, 2.60), the partially adjusted model (β: 2.26, 95% CI: 1.70, 2.82), and the fully adjusted model (β: 0.61, 95% CI: 0.06, 1.16). The results also suggested that PhenoAgeAccel was positively associated with depressive symptoms. In the fully adjusted model, participants with severe depressive symptoms had higher levels of PhenoAgeAccel (β: 1.48, 95% CI: 0.59, 2.37) compared to those with mild depressive symptoms (β: 0.56, 95% CI: 0.14, 0.98). A similar trend was observed in the unadjusted and partially adjusted models.

**Table 2:** Associations between depression and PhenoAgeAccel

PhenoAgeAccel (years)	No.	β (95% CI)		
		Model 1^a^	Model 2^b^	Model 3^c^
Major depression				
No	11,686	Reference	Reference	Reference
Yes	1,075	2.04 (1.48, 2.60) **	2.26 (1.70, 2.82) **	0.61 (0.06, 1.16) *
Depressive symptoms				
No	9,744	Reference	Reference	Reference
Mild	1,942	1.17 (0.74, 1.61) **	1.41 (0.97, 1.84) **	0.56 (0.14, 0.98) **
Moderate	697	1.65 (0.96, 2.33) **	1.91 (1.22, 2.59) **	0.34 (-0.33, 1.00)
Severe	378	3.32 (2.40, 4.24) **	3.62 (2.71, 4.54) **	1.48 (0.59, 2.37) **

Abbreviations: PhenoAgeAccel, phenotypic age acceleration

^a^ Model 1: adjusted for no covariates

^b^ Model 2: adjusted for age, gender, and race

^c^ Model 3: adjusted for age, gender, race, education, marital status, poverty income ratio, BMI, drinking status, smoking status, physical activity, and the history of hypertension, diabetes, cardiovascular disease, and cancer

*P < 0.05; **P < 0.01

**Fig. 1:**
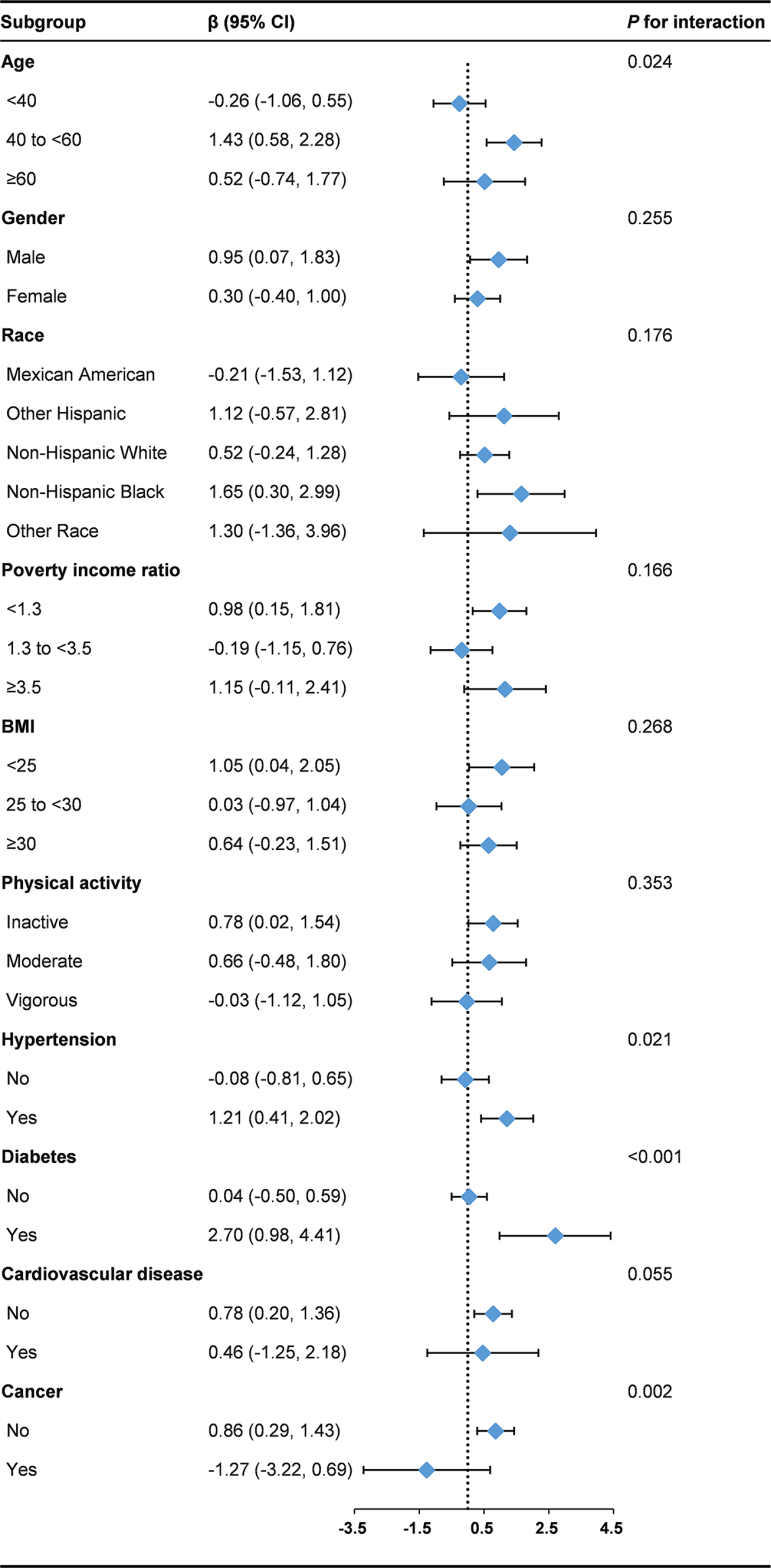
Subgroup analysis for the association between major depression and PhenoAgeAccel. The analysis adjusted for age, gender, race, education, marital status, poverty income ratio, BMI, drinking status, smoking status, physical activity, and the history of hypertension, diabetes, cardiovascular disease, and cancer, except for the stratification variable itself. Abbreviation: BMI, body mass index

The results of subgroup analyses are illustrated in Fig. 1. Significant interactions were observed between age, history of hypertension, history of diabetes, history of cancer, and major depression (*P* for interaction < 0.05). The association between major depression and PhenoAgeAccel was more statistically significant among participants aged 40 to < 60 years (β: 1.43, 95% CI: 0.58, 2.28), those with a history of hypertension (β: 1.21, 95% CI: 0.41, 2.02), those with a history of diabetes (β: 2.70, 95% CI: 0.98, 4.41), and those without a history of cancer (β: 0.86, 95% CI: 0.29, 1.43). None of the other variables significantly modified the association between major depression and PhenoAgeAccel (*P* for interaction > 0.05).

### Association between major depression and mortality outcomes

The median follow-up duration was 11.3 years (interquartile range, 9.9–13.1). During 141,636.6 person-years of follow-up, 1,971 all-cause deaths occurred, including 496 cardiovascular deaths and 471 cancer deaths. As shown in Table 3, major depression was associated with an increased risk of all-cause mortality in the unadjusted model (HR: 1.42, 95% CI: 1.17, 1.73), partially adjusted model (HR: 2.10, 95% CI: 1.72, 2.55), and fully adjusted model (HR: 1.35, 95% CI: 1.13, 1.62). A similar relationship was observed between major depression and cardiovascular mortality (Model 1, HR: 1.58, 95% CI: 1.09, 2.30; Model 2, HR: 2.61, 95% CI: 1.82, 3.74; Model 3, HR: 1.73, 95% CI: 1.18, 2.54). While major depression was significantly associated with increased cancer mortality in the partially adjusted model (HR: 1.75, 95% CI: 1.11, 2.74), this association was not observed in the unadjusted and fully adjusted models (Model 1, HR: 1.24, 95% CI: 0.80, 1.92; Model 3, HR: 1.19, 95% CI: 0.78, 1.82). As illustrated in Supplementary Fig. 2, none of these variables significantly altered the associations between major depression and all-cause, cardiovascular, or cancer mortality (*P* for interaction > 0.05).

**Table 3:** Associations between major depression and mortality outcomes

Mortality outcomes	Death/No.	Weighted death (%)	HR (95% CI)		
			Model 1^a^	Model 2^b^	Model 3^c^
All-cause mortality					
No major depression	1797/11,679	11.14	Reference	Reference	Reference
With major depression	174/1074	15.11	1.42 (1.17, 1.73) **	2.10 (1.72, 2.55) **	1.35 (1.13, 1.62) **
Cardiovascular mortality					
No major depression	454/11,679	2.67	Reference	Reference	Reference
With major depression	42/1074	4.04	1.58 (1.09, 2.30) *	2.61 (1.82, 3.74) **	1.73 (1.18, 2.54) **
Cancer mortality					
No major depression	429/11,679	2.69	Reference	Reference	Reference
With major depression	42/1074	3.21	1.24 (0.80, 1.92)	1.75 (1.11, 2.74) *	1.19 (0.78, 1.82)

Abbreviations: HR, hazard ratio

^a^ Model 1: adjusted for no covariates

^b^ Model 2: adjusted for age, gender, and race

^c^ Model 3: adjusted for age, gender, race, education, marital status, poverty income ratio, BMI, drinking status, smoking status, physical activity, and the history of hypertension, diabetes, cardiovascular disease, and cancer

*P < 0.05; **P < 0.01

### Mediated effect of PhenoAgeAccel on the association between major depression and mortality outcomes

**Fig. 2:**
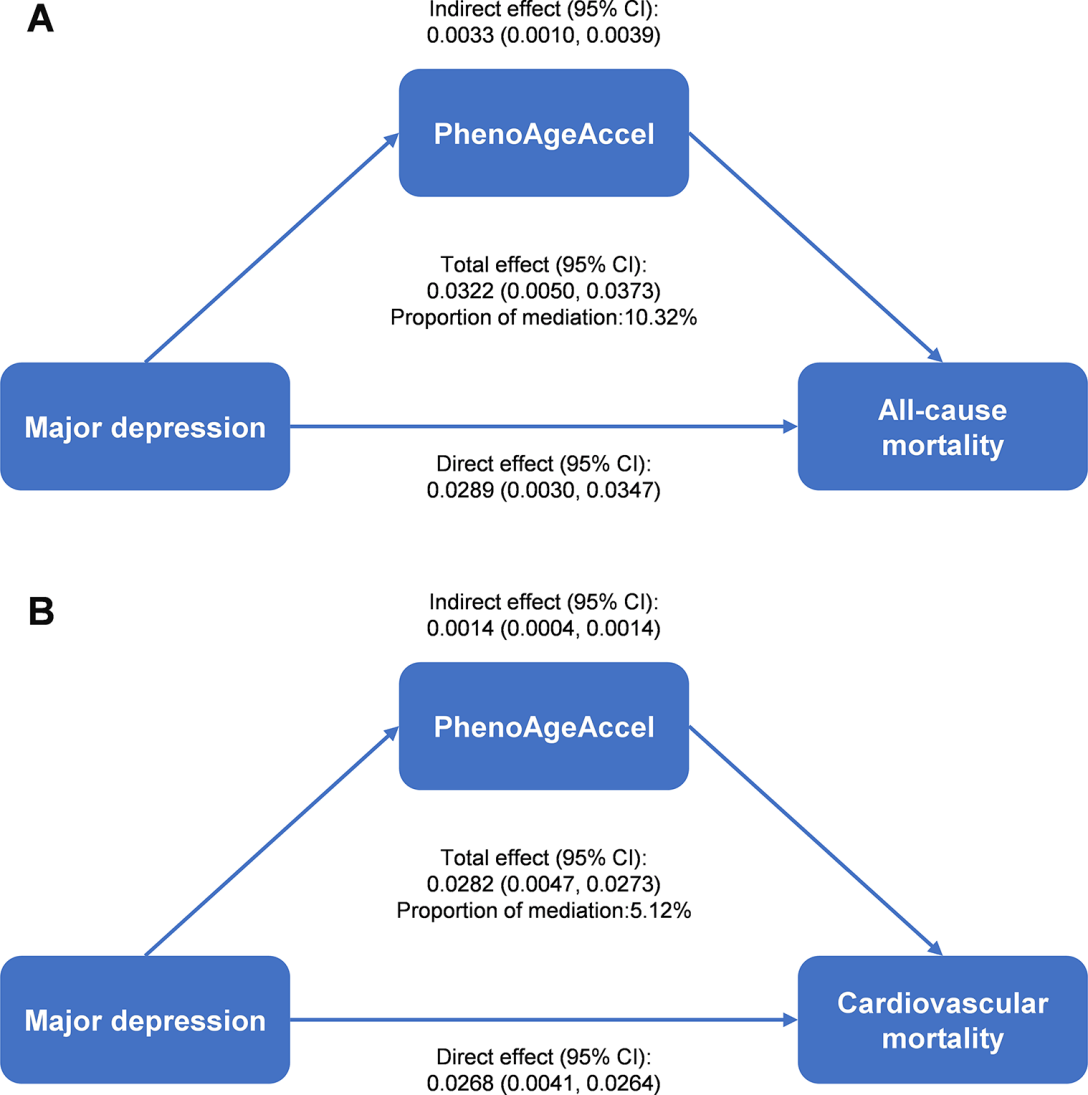
Mediated effect of PhenoAgeAccel on the association between major depression and (**A**) all-cause mortality and (**B**) cardiovascular mortality. This analysis quantified the total effect (association between major depression and mortality outcomes), the direct effect (total effect without the influence of PhenoAgeAccel), and the indirect effect (effect of major depression on mortality outcomes mediated by PhenoAgeAccel). The proportion of mediation refers to the percentage of the total effect that is explained by PhenoAgeAccel as the mediating variable in the association between major depression and mortality outcomes. Abbreviations: PhenoAgeAccel, phenotypic age acceleration

A mediation analysis was performed to assess the role of PhenoAgeAccel in mediating the association between major depression and mortality outcomes. As shown in Fig. 2 and Supplementary Table 1, PhenoAgeAccel mediated 10.32% of the association between major depression and all-cause mortality. In the analysis of the association between major depression and cardiovascular mortality, PhenoAgeAccel mediated 5.12% of the effect. However, the mediation effect of PhenoAgeAccel on the association between major depression and cancer mortality was not statistically significant.

## Discussion

In this large, nationally representative study of U.S. adults, we found that depression is associated with accelerated aging. Subgroup analysis revealed that this association is particularly significant among middle-aged participants, those with hypertension or diabetes, and those without cancer. Major depression was linked to an increased risk of all-cause and cardiovascular mortality, although its effect on cancer mortality was not statistically significant. Mediation analysis revealed that PhenoAgeAccel mediated 10.32% of the association between major depression and all-cause mortality, and 5.12% of the association with cardiovascular mortality. Our findings provide evidence to support integrating mental health care into public health strategies to delay population aging and reduce mortality risk.

Accumulating evidence suggests a link between mental health issues and accelerated biological aging. Previous studies have identified an association between depression and accelerated aging through various biomarkers, such as DNA methylation age [32], leukocyte telomere length [10, 33], and serum α-Klotho level [34]. A Mendelian randomization analysis has also provided causal evidence that depression contributes to the accelerated aging process [35]. However, several studies have failed to find an association between depression and accelerated aging [36, 37], which may be attributed to differences in study populations, definitions of depression, and biomarkers used to measure aging. Therefore, in this study, we included a large, representative sample from the NHANES, defining major depression and depressive symptoms using the self-reported PHQ-9 and measuring aging with PhenoAgeAccel. While some previous studies have found inconsistent results, this study found a positive association between depression and accelerated aging. However, subgroup analysis indicated that this relationship was not significant among older adults and women, which is in line with studies that did not observe an association between depression and accelerated aging [36, 37]. Additionally, significant interactions were observed between major depression and several chronic diseases, which is supported by prior research demonstrating relationships between chronic diseases and depression [38, 39]. These inconsistencies highlight the need for further research to explore the varying effects of depression on biological aging across different demographic groups.

Depression is not only linked to accelerated aging but also associated with an increased risk of mortality. Previous research has found that depression is associated with an increased risk of all-cause [40–43], cardiovascular diseases [13], and cancer mortality [44, 45]. Our study supports these findings, demonstrating that major depression significantly increases the risk of both all-cause and cardiovascular mortality. However, while there was a significant association between major depression and increased cancer mortality in the partially adjusted model, this association was not observed in the unadjusted and fully adjusted models. This inconsistency suggests that the observed relationship may be influenced by uncontrolled confounding factors. Cancer type and stage significantly influence mortality outcomes, and in studies focusing on specific types of cancer, the statistical association between depression and cancer mortality is not always observed [46–48]. Additionally, different forms of cancer therapies can substantially influence mental health and overall health outcomes [49–51]. Thus, future research should aim to comprehensively control for these variables to elucidate the true impact of depression on cancer mortality.

To our knowledge, this is the first study to investigate the mediating role of accelerated aging in the relationship between major depression and mortality risk. Previous studies have shown that accelerated aging mediates the relationship between unhealthy lifestyles and mortality risk [52]. Similarly, our study found that PhenoAgeAccel mediates the association between major depression and both all-cause and cardiovascular mortality outcomes. These findings may have important clinical implications. On the one hand, they provide valuable insights into the specific mechanisms by which depression increases mortality risk. On the other hand, they reveal a novel pathway for intervention, suggesting that strategies aimed at slowing biological aging may help mitigate the adverse health outcomes associated with depression.

Explaining the accelerated aging and increased mortality risk in patients with depression involves several potential biological mechanisms. Previous research has established a significant association between depression and elevated levels of the senescence-associated secretory phenotype (SASP) factors [53–55]. The SASP, produced by senescent cells, comprises a complex array of signaling proteins crucial for inflammatory control, tissue remodeling, cell growth, cell cycle regulation, and metabolic processes [56–58]. These factors operate synergistically across various tissues and cell types, promoting pro-senescence signals that induce cellular aging in both adjacent and distant cells [59, 60]. The systemic accumulation of senescent cells plays a pivotal role in accelerating the onset of age-related diseases and exacerbating clinical outcomes. In addition, mitochondrial dysfunction is a central contributor to both the pathophysiology of depression and the biological aging process [61]. Patients with depression exhibit elevated levels of reactive oxygen species (ROS), a key indicator of mitochondrial dysfunction [62, 63]. The increase in ROS induces oxidative stress, resulting in substantial damage to mitochondria, cells, and DNA, which elevates the risk of age-related diseases and contributes to increased mortality [64, 65]. More studies are necessary to clarify the mechanisms through which depression impacts biological aging, which will help identify new therapeutic targets and approaches to alleviate depression-associated aging and adverse health outcomes.

The strength of the study lies in its use of a large, nationally representative sample of the U.S. population from the NHANES. Despite its significant contributions, there are several limitations to consider. First, although we identified a positive association between depression and accelerated aging, the cross-sectional nature of the analysis prevents establishing causality. Second, the reliance on self-reported data for depression status and certain covariates introduces potential recall bias and inaccuracies. Finally, since NHANES is not a specialized database for cancer patients, it does not collect data on cancer stages and treatments, which may affect the analysis of cancer mortality outcomes.

## Conclusion

In this large, nationally representative study of U.S. adults, we found that depression is associated with accelerated aging and contributes to increased all-cause and cardiovascular mortality. Accelerated aging partially mediates the association between major depression and mortality risk. Our findings highlight the urgent need to incorporate mental health care into public health strategies to delay population aging and reduce mortality risk.

## Electronic supplementary material

Below is the link to the electronic supplementary material.

Supplementary Material 1

## Data Availability

The National Health and Nutrition Examination Survey dataset is publicly available at the National Center for Health Statistics of the Center for Disease Control and Prevention (https://www.cdc.gov/nchs/nhanes/index.htm).

## Abbreviations

BMI: Body mass index
CI: Confidence interval
HR: Hazard ratio
ICD-10: International Statistical Classification of Diseases 10th Revision
NHANES: National Health and Nutrition Examination Survey
PhenoAge: Phenotypic age
PhenoAgeAccel: Phenotypic age acceleration
PHQ-9: Patient Health Questionnaire-9
ROS: Reactive oxygen species
SASP: Senescence-associated secretory phenotype
