# Supplementary material for: Mediating role of accelerated aging in the association between depression and mortality risk: findings from NHANES
Source: Aging Clin Exp Res. 2024 Oct 5;36(1):202. doi: 10.1007/s40520-024-02854-z (PMC11455804; doi:10.1007/s40520-024-02854-z)
Supplement: Supplementary file 1 — Supplementary Material 1 [file 40520_2024_2854_MOESM1_ESM.pdf]

Supplementary material

Supplementary Methods 1 Calculations of PhenoAge and PhenoAgeAccel.

Supplementary Methods 2 The multivariable models for linear regression and Cox proportional hazards regression analysis.

Supplementary Table1 PhenoAgeAccel as mediators in the associations between depression and mortality outcomes.

Supplementary Figure 1 Flow chart of participants selection.

Supplementary Figure 2 Subgroup analysis for the association between major depression and (A) all-cause mortality, (B) cardiovascular mortality, and (C) cancer mortality.

Supplementary Methods 1 Calculations of PhenoAge and PhenoAgeAccel.

The resulting final equations for calculating PhenoAge and PhenoAgeAccel in this study are as follows:

$$\text{PhenoAge} = 141.50 + \frac{\ln[-0.00553 \times \ln(1 - \text{Mortality risk})]}{0.090165}$$

$$\text{PhenoAgeAccel} = \text{Phenotypic Age} - \text{Chronological Age}$$

Where:

$$\text{Mortality risk} = 1 - \exp\left(\frac{-1.51714 \times \exp(\text{xb})}{0.0076927}\right)$$

And:

$$\begin{aligned} \text{xb} = -19.907 - 0.0336 \times \text{albumin} + 0.0095 \times \text{Creatinine} + 0.1953 \times \text{Glucose} \\ + 0.0954 \times \ln(\text{C} - \text{reactive protein}) - 0.0120 \times \text{Lymphocyte Percent} \\ + 0.0268 \times \text{Mean Cell Volume} + 0.3306 \times \text{Red Cell Distribution Width} \\ + 0.00188 \times \text{Alkaline Phosphatase} + 0.0554 \times \text{White Blood Cell Count} \\ + 0.0804 \times \text{Chronological Age} \end{aligned}$$

Supplementary Methods 2 The multivariable models for linear regression and Cox proportional hazards regression analysis.

Three multivariate regression models were constructed:

Model 1: unadjusted.

Model 2: adjusted for age, gender, and race.

Model 3: adjusted for age, gender, race, education, marital status, poverty income ratio, BMI, drinking status, smoking status, physical activity, and the history of hypertension, diabetes, cardiovascular disease, and cancer.

Supplementary Table1 PhenoAgeAccel as mediators in the associations between depression and mortality outcomes.

Mortality outcome	Total effect (95% CI)	Indirect effect (95% CI)	Direct effect (95% CI)	Mediation (%)	*P* value
All-cause mortality	0.0322 (0.0050, 0.0373)	0.0033 (0.0010, 0.0039)	0.0289 (0.0030, 0.0347)	10.32	0.008
Cardiovascular mortality	0.0282 (0.0047, 0.0273)	0.0014 (0.0004, 0.0014)	0.0268 (0.0041, 0.0264)	5.12	0.010
Cancer mortality	0.0040 (-0.0043, 0.0134)	0.0009 (0.0002, 0.0009)	0.0031 (-0.0046, 0.0128)	21.54	0.416

The analysis adjusted for age, gender, race, education, marital status, poverty income ratio, BMI, drinking status, smoking status, physical activity, and the history of hypertension, diabetes, cardiovascular disease, and cancer. A *P* value <0.05 indicates that a statistically significant proportion of the total effect is mediated through the mediator.

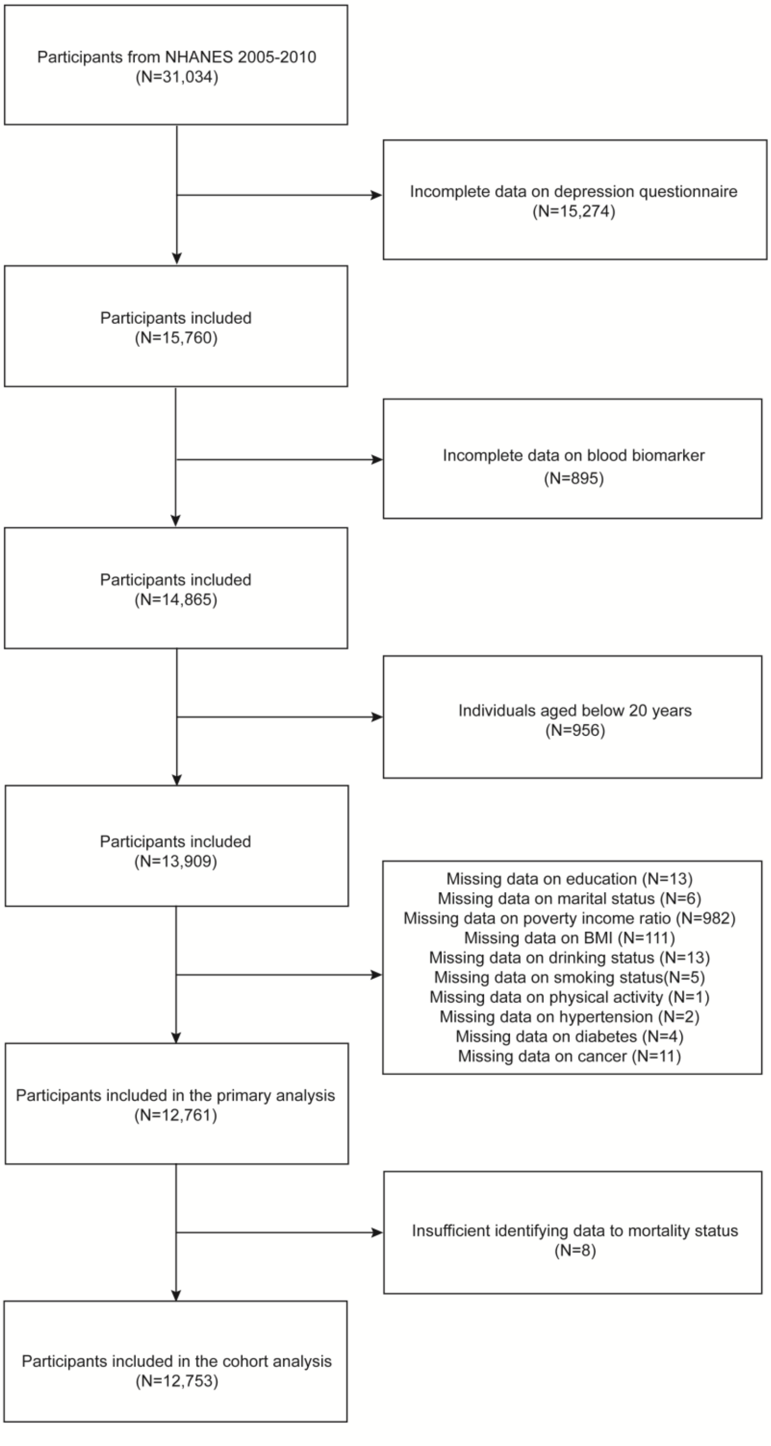

Supplementary Figure 1 Flow chart of participants selection.

Abbreviation: NHANES, National Health and Nutrition Examination Survey

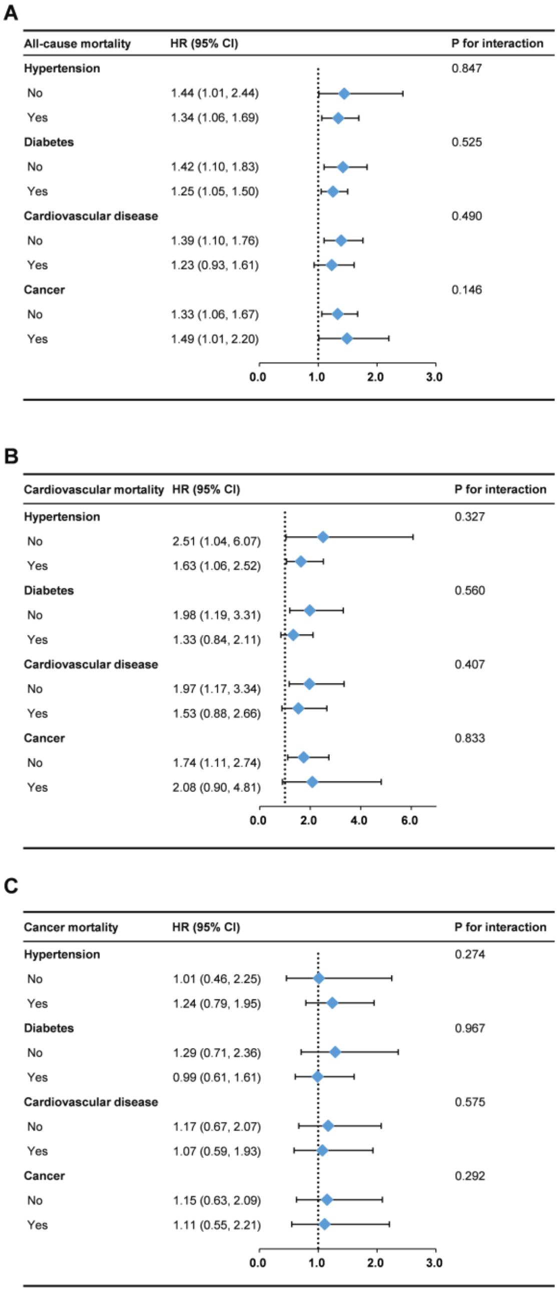

Supplementary Figure 2 Subgroup analysis for the association between major depression and (A) all-cause mortality, (B) cardiovascular mortality, and (C) cancer mortality.

The analysis adjusted for age, gender, race, education, marital status, poverty income ratio, BMI, drinking status, smoking status, physical activity, and the history of hypertension, diabetes, cardiovascular disease, and cancer, except for the stratification variable itself.
